# Expression of OPN3 in lung adenocarcinoma promotes epithelial‐mesenchymal transition and tumor metastasis

**DOI:** 10.1111/1759-7714.13254

**Published:** 2019-12-05

**Authors:** Chao Xu, Ruixia Wang, Yanfang Yang, Tongyi Xu, Yan Li, Jie Xu, Zhansheng Jiang

**Affiliations:** ^1^ Department of Breast Cancer, Tianjin Medical University Cancer Institute and Hospital, National Clinical Research Center for Cancer, Tianjin Key Laboratory of Cancer Prevention and Therapy Tianjin's Clinical Research Center for Cancer Tianjin China; ^2^ Department of Neurology The Second Hospital of Tianjin Medical University Tianjin China; ^3^ Thoracic and Cardiovascular Surgical Department, NO.971 Hospital of PLA Navy Qingdao China; ^4^ Department of Senior Ward, Tianjin Medical University Cancer Institute and Hospital National Clinical Research Center for Cancer,Tianjin Key Laboratory of Cancer Prevention and Therapy, Tianjin's Clinical Research Center for Cancer Tianjin China; ^5^ Department of Integrative Oncology, Tianjin Medical University Cancer Institute and Hospital, National Clinical Research Center for Cancer, Tianjin Key Laboratory of Cancer Prevention and Therapy Tianjin's Clinical Research Center for Cancer Tianjin China

**Keywords:** Epithelial‐mesenchymal transition, lung adenocarcinoma, metastasis, OPN3

## Abstract

**Background:**

Lung adenocarcinoma is the most common pathological lung cancer and an important cause of cancer‐related death. Metastasis is a major underlying reason for poor prognosis of lung adenocarcinoma. Opsin3 (OPN3), a member of the guanine nucleotide‐binding protein‐coupled receptor superfamily, has been identified to affect the apoptosis of hepatoma cells by modulating the phosphorylation of Akt and Bcl2/Bax. However, the expression and role of OPN3 in lung adenocarcinoma remains unclear.

**Methods:**

Opsin3 expression in lung adenocarcinoma tissues was detected by western blot, qPCR, and immunohistochemistry. Changes in cell migration and invasion ability resulting from the change of OPN3 expression level were detected by wound healing and transwell migration assays. Changes in the markers of epithelial‐mesenchymal transformation were detected by western blot and qPCR.

**Results:**

Opsin3 expression in lung adenocarcinoma tissues was higher than that in normal lung tissues. Patients with high expression of OPN3 had lower survival rates. Owing to overexpression of OPN3, the HCC827 cells showed enhanced invasion and migration ability in vitro. Upon decreasing the expression of OPN3, the invasion and migration ability of the A549 cells decreased.

**Conclusion:**

Our study demonstrated for the first time that OPN3 gene enhanced the metastasis in lung adenocarcinoma, and its overexpression promoted epithelial‐mesenchymal transition.

**Key points:**

A significant finding of the study was that OPN3 acted an oncogene in promoting lung adenocarcinoma metastasis. Our study complemented the research on the expression and function of OPN3 in lung adenocarcinoma.

## Introduction

Lung cancer is the most common cancer in the world and a leading cause of cancer‐related deaths.^1^, ^2^ Lung adenocarcinoma (LUAD) is the most prevalent type of pathological cancer.^3^ It accounts for up to 40% cases of lung cancer, with an average five‐year survival rate of approximately 15%.^4^, ^5^ However, for LUAD patients with distant metastasis, this rate is only about 1%.^6^, ^7^ Metastasis is a major underlying cause of a decreased survival rate in patients with LUAD,^8^ and cancer cells with high metastatic tendency often have widespread epithelial‐mesenchymal transition (EMT) characteristics.^9^, ^10^, ^11^ The EMT process is typically characterized by loss of epithelial markers (such as E‐cadherin) and increased mesenchymal markers (such as N‐cadherin, vimentin), which promotes tumor cells to invade and metastasize. Therefore, it is critical to further explore the underlying genes and mechanism in tumor metastasis for the treatment of LUAD.

Opsin3 (*OPN3*), first discovered in brain tissue,^12^ is located on chromosome 1q43. It is a member of the guanine nucleotide‐binding protein (G‐protein)‐coupled receptor superfamily.^13^ The OPN3 encodes for a transmembrane protein that contains seven α‐helix transmembrane domains, with C‐terminus rich in serine and threonine, and a glycosylated N‐terminus.^13^ Opsin3 gene is highly expressed in the brain and testis; however, low expression is seen in normal lung tissue.^13^ Previous studies have demonstrated that OPN3 is expressed in lung bronchial epithelial cells and may be associated with asthma.^14^ It can affect the apoptosis of hepatoma cells by regulating phosphorylation of Akt and Bcl2/Bax.^15^ Previous studies have also confirmed the expression of *OPN3* in normal lung tissue; however, to date there is no relevant study on its expression and function in LUAD.

## Methods

### Patients

The tissue samples were obtained from 114 LUAD patients admitted to Tianjin Medical University Cancer Hospital (Tianjin,China) between 2010 and 2015. The use of patient tissue specimens and clinicopathological data was approved by the Tianjin Medical University Cancer Institute and the Hospital Ethics Committee.

### Cell lines, cell culture and treatment

The LUAD cell lines, A549, HCC827, NCI‐H1975, NCI‐H522, NCI‐H23 and normal cell line BEAS‐2B were stored in liquid nitrogen at the Institute of Oncology. Cell lines A549, HCC827, NCI‐H522, NCI‐H23 and NCI‐H1975 were cultured using RPMI‐1640 medium containing 10% fetal bovine serum in a humidified incubator at 37°C containing 5% CO_2_. The OPN3 overexpression plasmid (CMV‐3FLAG) was purchased from GeneChem (Shanghai, China) and SiRNA that reduces the expression of OPN3 was purchased from RiboBio (Guangzhou, China). Plasmid and SiRNA transfection was performed using Lipofectamine 2000 (Invitrogen, Carlsbad, CA, USA) as per the instructions of the manufacturer.

### Immunohistochemistry

Immunohistochemistry was carried out as per the procedure described elsewhere.^16^ The OPN3 antibody (abcam, ab228748, USA) was used as a primary antibody. Immunohistochemical staining score was calculated according to the area and intensity of the positive staining field of tumor tissues. The scoring standard was utilized from the article by Hao *et al*.^17^ Immunohistochemical staining intensity was categorized as 0 (negative), one (low expression), two (medium expression), and three (high expression). Staining degree was divided as 0 (0% staining), one (1%–25% staining), two (26%–50% staining), and three (51%–100% staining). The final score was calculated by multiplying the staining intensity with the staining degree. It was defined as negative (<2 points), low expression (2–3 points), moderate expression (4–6 points), and high expression (>6 points).

### Real‐time quantitative polymerase chain reaction

Total RNA extracted from LUAD cell lines and tissues was treated with TRIzol reagent (TaKaRa, China). Primer sequences for OPN3 were referenced to the sequence listed in Jiao *et al*.^15^ Primer sequences (for *CDH1, CDH2*, *Vimentin, Slug, Snail*) and the experimental procedures were used as described in Xu *et al*.^16^

### Western blotting

The total protein from tumor cells and tissues was extracted by lysis using SDS Lysis Buffer. The OPN3, CDH1 (CST, 14472s, USA), CDH2 (CST, 13116s, USA), Vimentin (CST, 5741, USA), Snail (CST, 3879s, USA), Slug (CST, 9585s, USA) and β‐Tubulin (Ray antibody, RM2003, China) were used as primary antibodies. The proteins of the same mass were separated by SDS‐page gel electrophoresis and then transferred to Polyvinylidene fluoride (PVDF) membrane (Merk, Germany) by constant current (260 mA) for two hours. The PVDF membrane was sealed with 5% milk at 25°C for two hours, and then soaked in a primary antibody solution of appropriate concentration for 4°C overnight. The PVDF membrane was then washed the next day three times with TBST and immersed in HRP‐conjugated secondary antibody solution (Ray antibody Biotech, Beijing, China) for two hours at room temperature. After the PVDF membrane had been washed three times with TBST, fluorescence enhancer ECL (Merk, Germany) was added for development. The photos were taken by Tanon 6600 luminescent imaging station (Tanon, China).

### Wound healing assay

In the wound healing experiment, 8 × 10^5^ cells were evenly spread on a six‐well plate. When the cell proliferation reached a density of approximately 80%, the monolayer cells were scratched using a 10 uL pipette tip and then cultured in serum‐free medium. Cell migration was recorded by microscopy at 0 and 24th hour. This experiment was independently repeated three times.

### Transwell migration and invasion assays

Invasion and migration tests were performed using Corning's chamber (Corning, NY, USA). The specific experimental steps were followed as described elsewhere.^16^ Cells passing through the basement membrane of the upper chamber were stained with a three‐step staining kit (ThermoFisher Scientific, USA), and statistical analysis of four random field counts was then performed under a 200x microscope. Invasion and migration experiments were independently repeated three times.

### Statistical analysis

Statistical analyses were performed using SPSS 22.0 (SPSS Inc., Chicago, Illinois, USA) and GraphPad Prism software (La Jolla, CA, USA). The Wilcoxon signed rank test was used to statistically compare the difference of OPN3 expression in LUAD tissues and normal lung tissues. A chi‐square test was used to analyze the relationship between OPN3 expression level and clinicopathological data of LUAD patients. A Log rank test was used to analyze the overall survival and disease‐free survival of LUAD patients with different OPN3 expression levels. Gene set enrichment analysis (GSEA)^18^, ^19^ was used to analyze the OPN3 high expression group and OPN3 low expression group data selected from the TCGA data in LUAD cancer. Statistical significance was set at *P <* 0.05.

## Results

### Overexpression of OPN3 in lung adenocarcinoma

**Figure 1:**
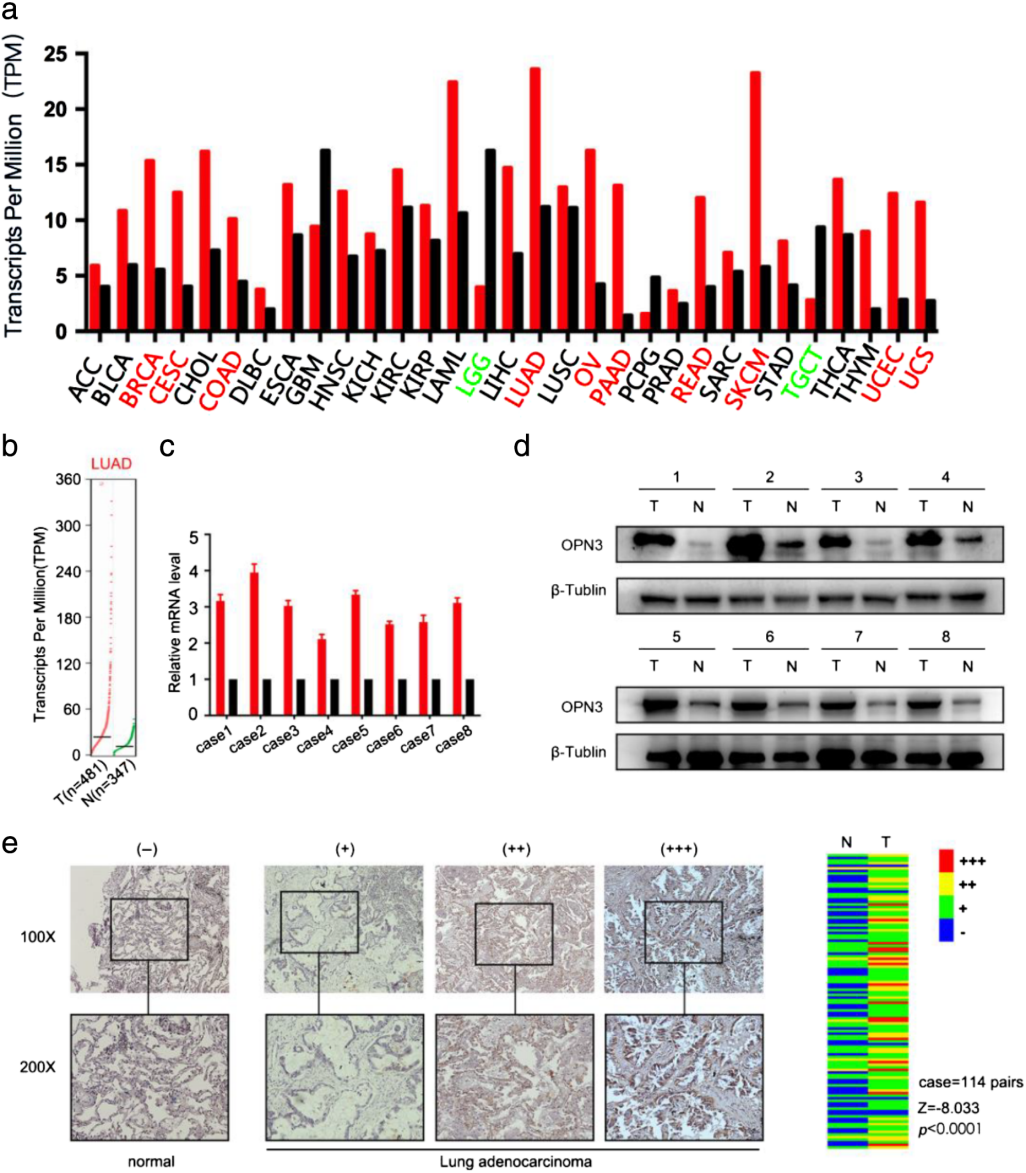
Overexpression of OPN3 in lung adenocarcinoma. (**a**) The OPN3 expression profile across all tumor samples and paired normal tissues. The height of each bar represents the median expression of a certain tumor type or normal tissue. The red‐labeled tumors indicate that the expression of OPN3 in cancer tissues is higher than that in normal tissues, and the green‐labeled tumors indicate that OPN3 expression in cancer tissues is lower than that in normal tissues (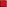) Tumor, and (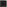) Normal. (**b**) The expression of OPN3 in LUAD tissues of TCGA data was compared with the expression of OPN3 in normal lung tissues using GTEx data. The results were statistically different. (**c** and **d**) The expression of OPN3 in eight pairs of fresh LUAD tissues and corresponding adjacent tissues was detected by qPCR and western blot (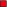) T, and (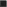) N. (e) The expression of OPN3 in cancer tissues and paired paracancer tissues of 114 patients with LUAD, from left to right: normal paracancer tissues, low, medium and high expression in cancer tissues, respectively. The heat map on the right shows the immunohistochemical score of OPN3 expression in paired paracancer and tumor tissues of 114 patients with LUAD. The Wilcoxon signed‐rank test was employed, and the results were statistically different. LUAD, lung adenocarcinoma; T, tumor tissues; N, normal tissues; *P < 0.05.

With TCGA data analysis, we found that the expression of OPN3 in LUAD and other cancers (breast cancer, cervical cancer, colon adenocarcinoma, ovarian serous cystadenocarcinoma, pancreatic adenocarcinoma, rectal adenocarcinoma, skin cutaneous melanoma, uterine corpus endometrial carcinoma, uterine carcinosarcoma, Fig 1a,b, *P <* 0.05) was higher than in the corresponding normal tissues. To verify this analysis, we collected eight pairs of cancer tissues and adjacent normal tissues from LUAD patients who had undergone surgery and conducted qPCR (Fig 1c) and western blot experiments (Fig 1d). The results indicated that the expression of OPN3 in LUAD cancer tissues was higher than that in normal lung tissues in both these experiments. Next, we confirmed OPN3 overexpression in LUAD tissues by performing immunohistochemical staining in 114 pairs of cancer and adjacent tissues of LUAD patients. The Wilcoxon signed rank test analysis showed that OPN3 expression was statistically different between the two groups (Fig 1e, *P* < 0.0001). Taken together, both TCGA analysis and experimental results confirmed that the expression of OPN3 was elevated in LUAD patients.

### Overexpression of OPN3 associated with poor prognosis of patients with LUAD and cancer metastasis promotion

**Figure 2:**
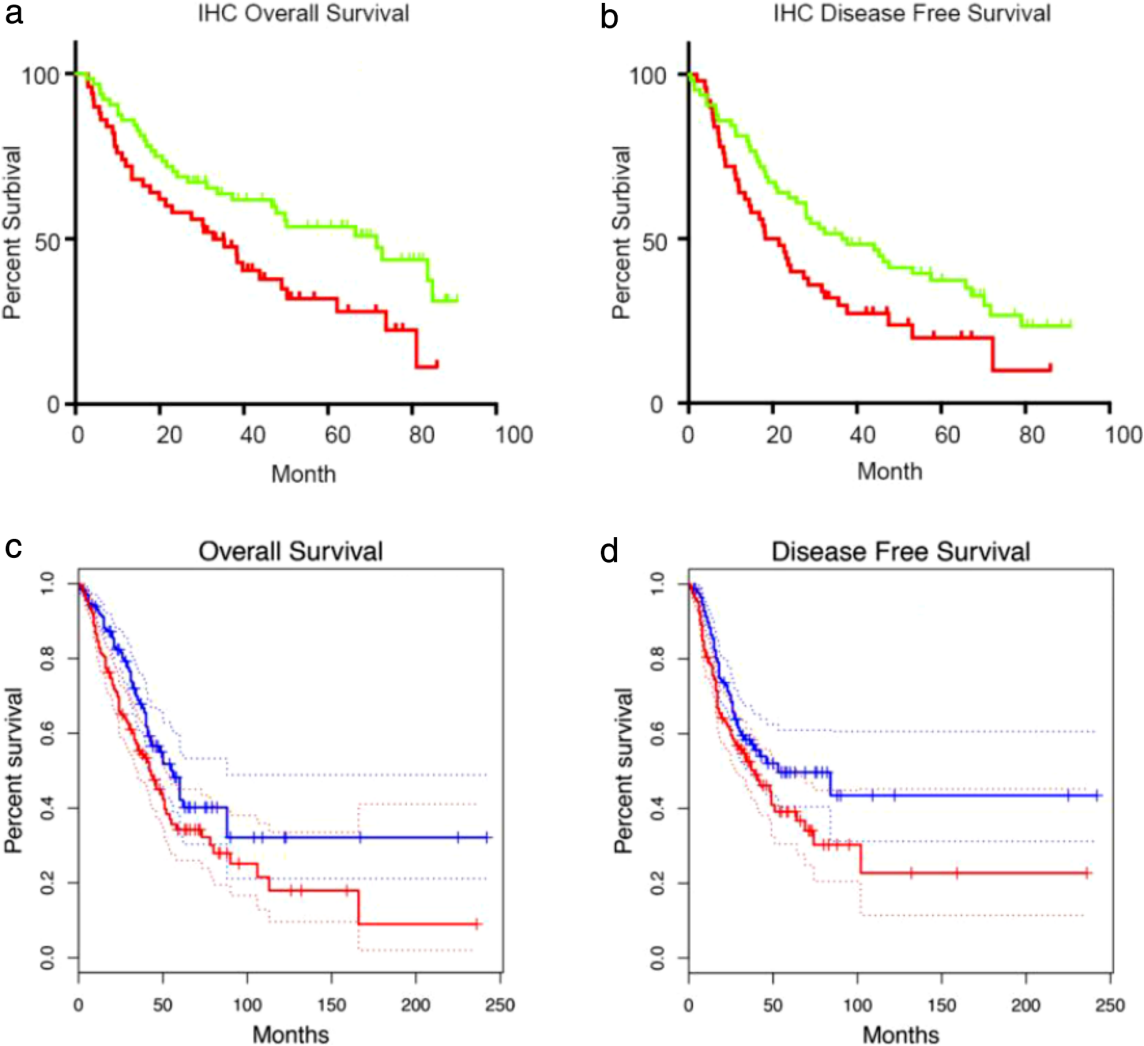
Survival analysis of patients with high expression of OPN3 and low expression of OPN3 in LUAD. (**a** and **b**) Comparison of overall survival and disease‐free survival between the OPN3 high‐ and low‐expression group in 114 patients with LUAD by Kaplan‐Meier analysis (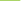) low OPN3, and (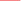) high OPN3. (**c** and **d**) Differences in overall survival and disease‐free survival between OPN3 high‐ and low‐ expression group in TCGA data of patients with LUAD were detected by Kaplan‐Meier analysis (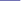) low OPN3 TPM, and (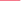) high OPN3 TPM. log‐rank test, *P* < 0.05.

Lung adenocarcinoma patients were divided into two groups for survival analysis. According to the OPN3 IHC staining score, it was found that the overall survival (OS) and disease‐free survival (DFS) of the OPN3 high‐expression group was significantly shorter than the OPN3 low‐expression group (Fig 2a,b, *P <* 0.05). The clinicopathological data showed that the expression of OPN3 was correlated with the lymph node metastasis in LUAD patients (Table 1). Analysis of TCGA data revealed the same trend of OPN3 being negatively correlated with OS and DFS (Fig 2c,d) and directly correlated with lymph node metastasis in LUAD patients (Table 2).

**Table 1:** Relationship between the level of OPN3 expression and clinical features in patients with LUAD

	OPN3 level			
	Low (*n* = 64)	High (*n* = 50)	χ2	*P*‐value
Gender			0.162	0.687
Male	36	30		
Female	28	20		
Age (year)			2.019	0.155
<65	38	23		
≥65	26	27		
pT			0.347	0.556
T1–T2	51	42		
T3–T4	13	8		
pN			5.344	0.021*
N0	50	29		
N1–N2	14	21		
pTNM			3.577	0.059
I	42	24		
II–III	22	26		

* *P* < 0.05 (Chi‐square tests).

**Table 2:** Relationship between the level of OPN3 mRNA expression and clinical features in patients with LUAD from TCGA data

		OPN3 level			
	Total	Low	High	χ2	*P*‐value
Gender				2.042	0.153
Male	238	126	112		
Female	277	164	113		
Age (year)				4.662	0.031*
<65	220	111	109		
≥65	276	166	110		
T staging				0.039	0.843
T1–T2	446	251	195		
T3–T4	66	38	28		
Nodal staging				6.18	0.013*
N0	331	200	131		
N1–N3	172	84	88		
M staging				2.172	0.141
M0	484	276	208		
M1	26	11	15		
TNM				2.497	0.114
I–II	397	232	165		
III–IV	110	55	55		

* *P* < 0.05 (Chi‐square tests).

### Overexpression of OPN3 enhances invasion and migration of LUAD cells

**Figure 3:**
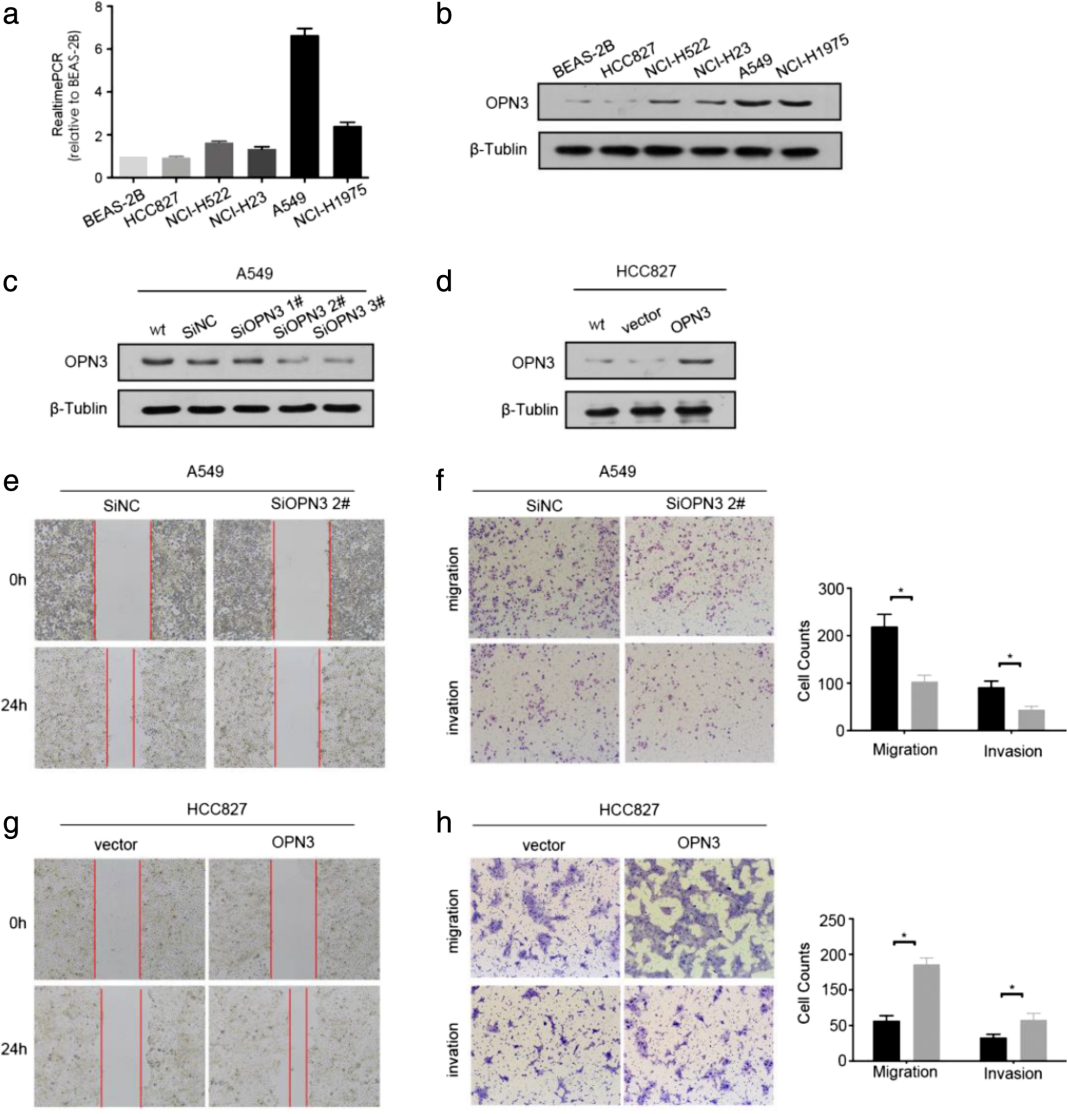
The expression of OPN3 affects the migration and invasion of LUAD cells. (**a** and **b**) The basic endogenous expression of OPN3, including RNA and protein levels, in normal lung epithelial cell line (BEAS‐2B) and five lung adenocarcinoma cell lines (A549, HCC827, NCI‐H522, NCI‐H23, NCI‐H1975). (**c** and **d**) The expression of OPN3 in A549 cell lines was decreased by transfecting OPN3 siRNA (siOPN3 1#,2#,3#), and the expression of OPN3 in HCC827 cell lines was increased by transfecting OPN3 overexpressed plasmid, which was verified by western blot. (**e**) Wound healing experiments were performed in the A549 cells transfected with negative control siRNA (SiNC) and siOPN3 2# for 24 hours. (**f**) Transwell migration and invasion assays were performed in the A549 cells transfected with negative control siRNA (SiNC) and siOPN3 2#, the counts of cell passing through the chamber between the two groups were statistically different (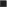) SiNC, and (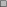) SiOPN3 2#. (**g**) Wound healing experiments were performed in the HCC827 cells transfected with pcDNA3.1(vector) and pcDNA3.1‐OPN3 for 24 hours. (**h**) Transwell migration and invasion assays were performed in the HCC827 cells transfected with pcDNA3.1(vector) and pcDNA3.1‐OPN3, the counts of cell passing through the chamber between the two groups was statistically different (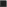) vector, and (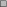) OPN3. *P < 0.05.

For our in vitro experiments, we first detected the basic expression levels of OPN3 in LUAD cell lines HCC827, A549, NCI‐H522, NCI‐H23 and NCI‐H1975 as well as normal lung epithelial cell lines BEAS‐2B by qPCR and western blot (Fig 3a,b). In the A549 cell line, the OPN3 expression was relatively high and it decreased with transient transfection of siOPN3, which was verified by western blot (Fig 3c). Of the three pairs of siRNAs, siRNA 2# most effectively reduced OPN3 expression and was selected for subsequent experimental studies. The wound healing and transwell migration assays (Fig 3e,f), confirmed that the migration and invasion of A549 cells were weakened after downregulation of OPN3 was. The HCC827 cell line had relatively low expression of OPN3 (Fig 3a,b), which increased by transient transfection of the OPN3 overexpression plasmid (Fig 3d), thereby enhancing its cell migration and invasion abilities (Fig 3g,h). This suggests that overexpression of OPN3 enhances the migration and invasion ability of LUAD cells.

### OPN3 promotes LUAD metastasis by inducing epithelial‐to‐mesenchymal transition

**Figure 4:**
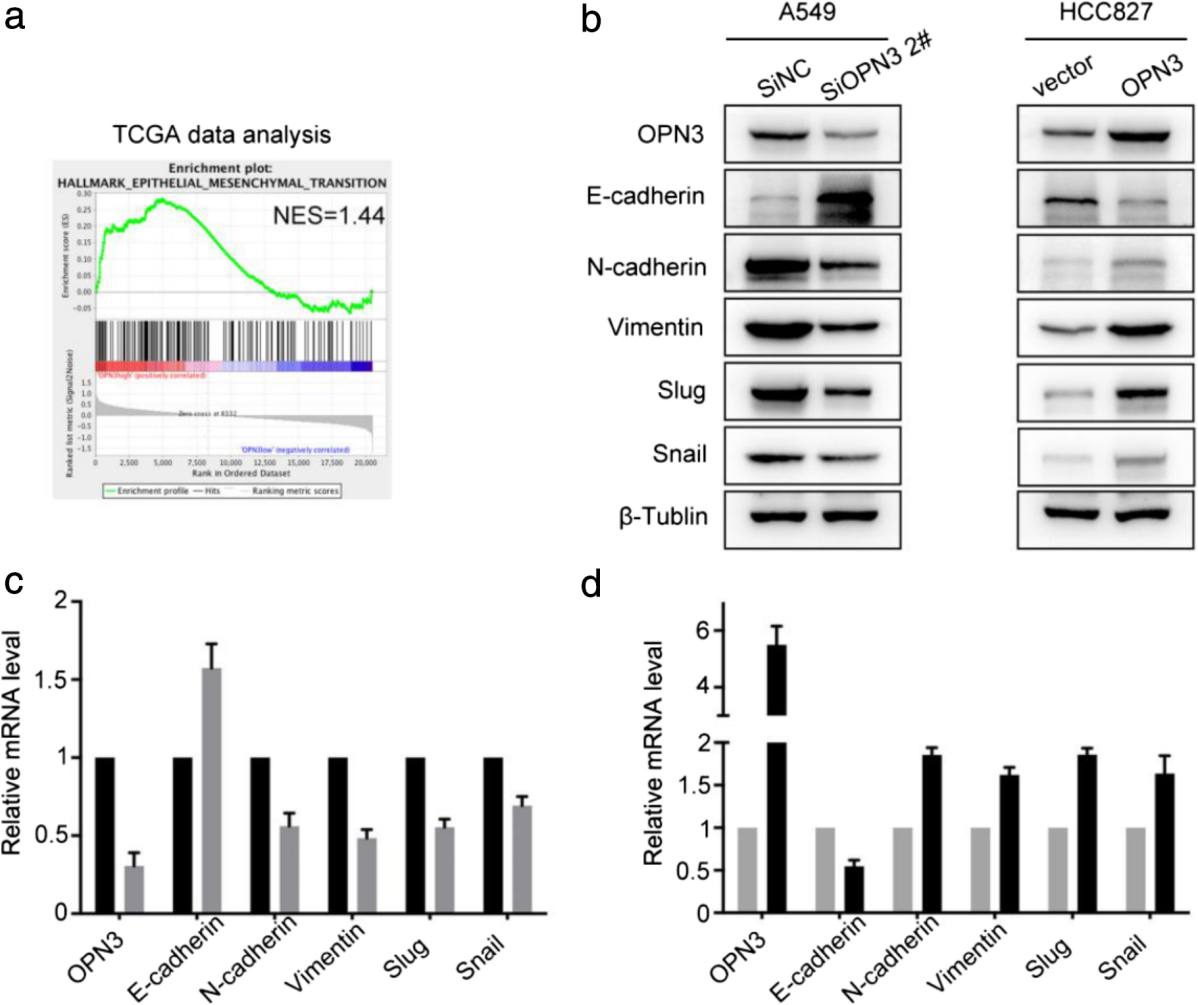
Expression of OPN3 affects epithelial‐mesenchymal transition (EMT) in LUAD cells. (**a**) The GSEA analysis was performed on TCGA data for LUAD; NES: normalized enrichment score. (**b**) EMT‐related marker changes were verified by western blot and qPCR in A549 cells transfected with negative control siRNA (SiNC) and SiOPN3, and in HCC827 cells transfected with pcDNA3.1 (vector) and pcDNA3.1‐OPN3. (**c**) (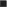) SiNC, and (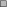) SiOPN3 2#. (**d**) (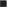) vector, and (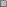) OPN3.

Gene set enrichment analysis (GSEA) was performed on TCGA data of LUAD patient (10 OPN3 high‐expression samples and 10 OPN3 low ‐expression samples).^20^ The GSEA analysis suggested that changes in OPN3 expression were associated with EMT in LUAD patients (Fig 4a). To verify this finding, changes in the epithelial markers (E‐cadherin), mesenchymal markers (N‐cadherin and Vimentin) and EMT‐related transcription factors (Snail and Slug) were compared using western blot and qPCR in A549 and HCC827 cell line. The results of both these assays showed the same trend. In the A549 cell line, with the downregulation of OPN3 expression, the expression of E‐cadherin increased, while the expression of N‐cadherin, Vimentin, Snail and Slug decreased (Fig 4b,c). In HCC827 cell lines, the expression of N‐cadherin, Vimentin, Snail and Slug increased while that of E‐cadherin decreased with the overexpression of OPN3 (Fig 4b,c). Therefore, the changes in OPN3 expression were associated with the EMT process, which may contribute to tumor metastasis in LUAD.

## Discussion

Lung adenocarcinoma is the most common pathological type of lung cancer and metastasis is the main cause for its poor prognosis. Therefore, there is an urgent need to explore new transfer‐driven genes and potential molecular mechanisms to formulate targeted therapeutic strategies to improve LUAD treatment.

In this study, we demonstrated that OPN3, a G‐protein‐coupled receptor, is related to the metastasis and clinical prognosis of LUAD. To date, only a few studies have reported its role in cancer. Only one article has previously reported that OPN3 might affect apoptosis of hepatoma cells by modulating the phosphorylation of Akt and Bcl2/Bax.^15^ We demonstrated that OPN3 could promote the EMT and tumor metastasis in LUAD. It has been previously reported that a variety of G‐protein coupled‐receptor proteins promote metastasis by affecting the EMT process of cancer cells,^21^, ^22^, ^23^, ^24^, ^25^, ^26^ including in lung cancer.^27^, ^28^, ^29^ However, more studies are warranted to further explore the molecular mechanism of OPN3 in affecting the EMT of LUAD.

In our study, we also demonstrated that the expression of OPN3 in LUAD cancer tissues was significantly higher than that in adjacent normal lung tissues. The LUAD patients with high expression of OPN3 had poor survival and were more prone to lymph node metastasis.

In conclusion, in our study we revealed for the first time that OPN3 acts as a gene that enhances metastasis, and overexpression promotes EMT in LUAD cells. The results of our study also suggest that OPN3 could be a clinically useful prognostic indicator for LUAD patients and may potentially become a new target for LUAD‐targeted therapy.

## Disclosure

The authors declare there are no potential conflicts of interest.
